# The role of transient receptor potential channels in chronic kidney disease-mineral and bone disorder

**DOI:** 10.3389/fphar.2025.1583487

**Published:** 2025-05-09

**Authors:** Lerong Zhang, Penghao Xu, Lele Hao, Lingling Wang, Yunkai Xu, Chen Jiang

**Affiliations:** ^1^ Department of Nephrology, First Teaching Hospital of Tianjin University of Traditional Chinese Medicine, Tianjin, China; ^2^ National Clinical Research Center for Chinese Medicine Acupuncture and Moxibustion, Tianjin, China; ^3^ Department of Biostatistics, The University of Alabama at Birmingham, Birmingham, AL, United States

**Keywords:** chronic kidney disease-mineral and bone disorder, transient receptor potential channels, mineral metabolism disorders, renal osteodystrophy, vascular calcification

## Abstract

Chronic kidney disease (CKD) represents a major global health challenge, frequently resulting in the development of chronic kidney disease-mineral and bone disorder (CKD-MBD). Transient receptor potential (TRP) channels, particularly the TRPV (vanilloid), TRPC (canonical), and TRPM (melastatin) subfamilies, are crucial in CKD-MBD by regulating calcium homeostasis, bone remodeling, and vascular calcification. Pharmacological agents targeting TRP channels and traditional Chinese medicine therapies demonstrate promising therapeutic potential for CKD-MBD. This article explores the role of TRP channels in CKD-MBD, from molecular mechanisms to treatment prospects, aiming to provide new insights for CKD-MBD treatment.

## 1 Introduction

**FIGURE 1:**
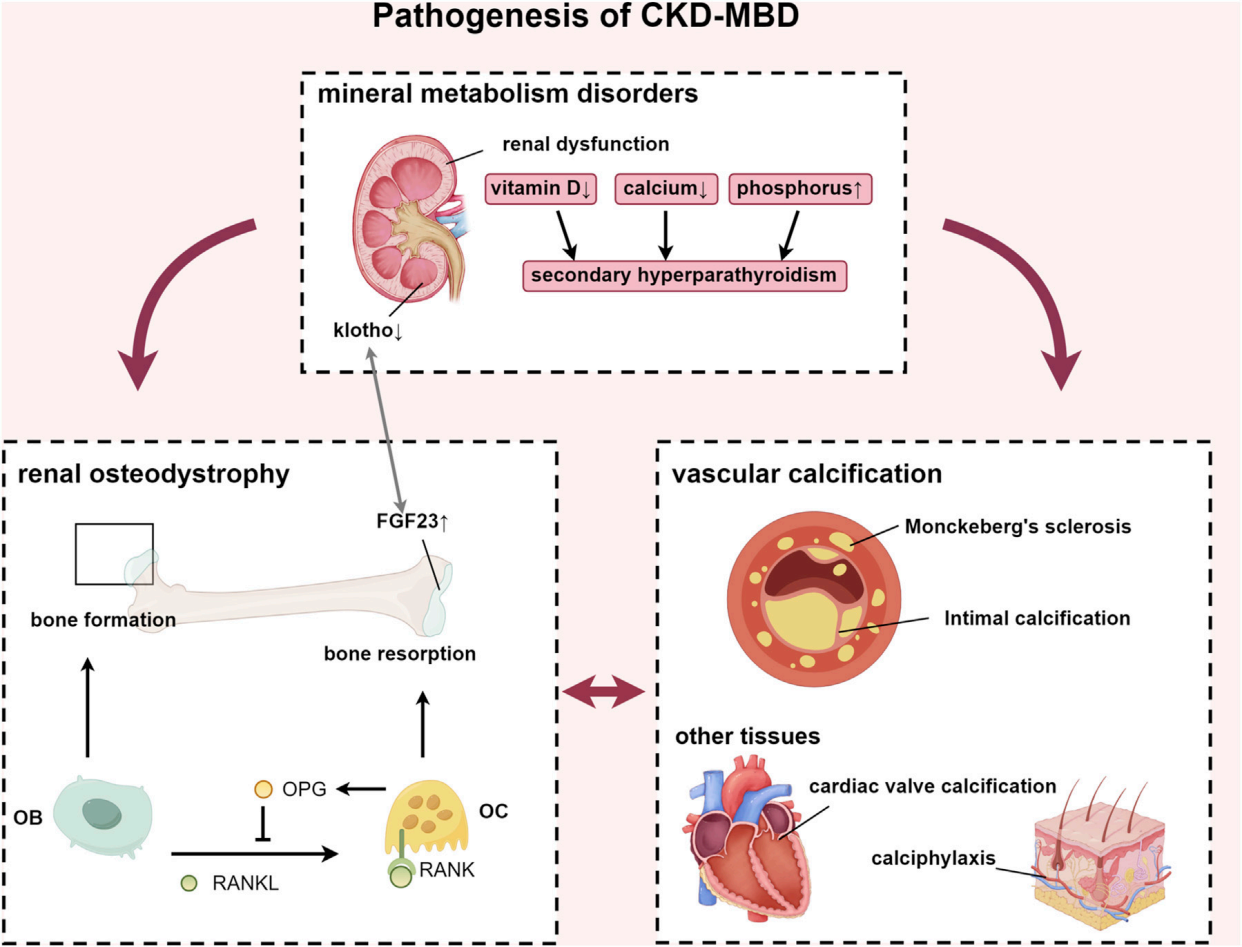
Pathogenesis of CKD-MBD. Abbreviations: FGF23, fibroblast growth factor 23; RANKL, receptor activator of nuclear factor-κB ligand; RANK, receptor activator of nuclear factor-κB; OPG, osteoprotegerin; OB, osteoblast; OC, osteoclast.

CKD represents a significant global health challenge, affecting approximately 9.1% of the population (GBD Chronic Kidney Disease Collaboration, 2020). Projections indicate that by 2040, CKD will become the fifth leading cause of life years lost worldwide (Foreman et al., 2018). CKD-MBD is a systemic disorder of mineral and bone metabolism caused by CKD. According to KDIGO’s authoritative definition, the diagnostic criteria for CKD-MBD include: (1) laboratory abnormalities: calcium, phosphorus, PTH, or vitamin D metabolism; (2) bone disease affecting bone turnover, mineralization, volume, linear growth, or strength; (3) vascular or other soft tissue calcification. One or more combinations of the above can be defined as CKD-MBD (Moe et al., 2006) (Figure 1). Among patients with end-stage renal disease (ESRD), the prevalence of CKD-MBD is as high as 86% (Magagnoli et al., 2023), significantly impairing patients’ quality of life, and elevating the incidence of cardiovascular disease and mortality risk. The pathogenesis of CKD-MBD is intricate. Typically, it is associated with disturbances in calcium and phosphorus metabolism, hyperparathyroidism, vitamin D deficiency, and the increase in serum fibroblast growth factor 23 (FGF23) levels (Kidney Disease: Improving Global Outcomes KDIGO CKD-MBD Update Work Group, 2017).

In recent years, numerous studies have demonstrated the significant role of TRP channels in the pathogenesis and progression of CKD-MBD. The TRP superfamily comprises a group of non-selective cation channel proteins that are extensively distributed across cell membranes and can be categorized into seven distinct subfamilies. These subfamilies include TRPV, TRPA, TRPC, TRPM, TRPN, TRPP, and TRPML. Each of these channels is characterized by six transmembrane domains, along with intracellular N-terminal and C-terminal domains, and they form a pore structure composed of hydrophobic groups between the fifth and sixth transmembrane domains, facilitating ion permeation. This structural configuration allows TRP channels to respond to a diverse range of stimuli. Unlike many voltage-gated ion channels, TRP channels lack a fully intact negatively charged residue in their fourth transmembrane domain. They mainly function as receptor-operated and store-operated Ca^2+^ channels, facilitating Ca^2+^ influx. Therefore, they are typically categorized as Ca^2+^ channels. Recent studies have demonstrated that the function of TRP channels extends significantly beyond their initially identified roles in sensory processes such as vision, olfaction, gustation, audition, thermosensation, nociception, pruritus, and certain reflexive responses (Menè et al., 2013). Patients with CKD-MBD exhibit a complex array of pathophysiological conditions, including metabolic acidosis, oxidative stress, chronic inflammation, apoptosis, and other microenvironmental alterations. These conditions contribute to the dysregulation of TRP channel activity, which in turn exacerbates the progression of CKD-MBD. Consequently, it is imperative to explore the involvement of TRP channels in CKD-MBD as a novel research focus.

## 2 The origin of imbalance: TRP channels and mineral metabolism disorders

### 2.1 Mineral metabolism disorders

Abnormal mineral metabolism represents the initial manifestation of CKD-MBD and serves as the initiating factor for a cascade of severe complications associated with CKD-MBD. The combination of hyperphosphatemia, hypocalcemia, and vitamin D deficiency contributes to the development of secondary hyperparathyroidism (SHPT). Increased parathyroid hormone (PTH) levels mobilize calcium from bone into the bloodstream, exacerbating bone loss and perpetuating a detrimental cycle.

### 2.2 TRPV5 and TRPV6: regulators of calcium homeostasis

TRPV5 and TRPV6, part of the TRPV subgroup with 75% amino acid homology, are capable of forming heterotetrameric channels. These channels are activated through the binding of phosphatidylinositol 4,5-bisphosphate (PI(4,5)P_2_) at the Arg302 and Lys484, and inhibited by calcimimetics. They are also activated by 1,25-dihydroxyvitamin D, PTH, and 17β-estradiol, but inhibited by acidosis. Patients with CKD accumulate non-volatile acids and have impaired acid excretion, leading to high anion gap metabolic acidosis. The resultant low pH narrows the TRPV5 channel’s ion pathway and stabilizes its closed state by forming salt bridges between Lys607 and Asp406, as well as between Arg409 and Glu294, that inhibit PI(4,5)P_2_ binding (Fluck et al., 2022). Consequently, this reduces calcium reabsorption in the distal tubule and increases urinary calcium excretion. Known as epithelial calcium channels, TRPV5 and TRPV6 are the most calcium-selective TRP channels, with a calcium-to-sodium selectivity ratio (PCa/PNa) over 100 due to Asp542 or Asp541 (Diver et al., 2022). They respond to low intracellular Ca^2+^ levels but are inhibited by elevated Ca^2+^ concentrations near their openings to prevent excessive Ca^2+^ influx (Bokhovchuk et al., 2018; Vangeel and Voets, 2019). These channels are distributed across various calcium-transporting tissues, including the kidney, intestines, bone, pancreas, prostate, and placenta. TRPV5 is mainly found in the distal convoluted tubule (DCT) and connecting tubule (CNT) of the kidney, with a particular abundance in the DCT2 segment (Peng et al., 2018), while TRPV6 is primarily located on the brush border of small intestinal cells and is also present in the kidney and bone (Khattar et al., 2022). Calcium functions as a critical second messenger, playing an essential role in bone health, muscle contraction, nerve signaling, and apoptosis. Its systemic metabolism is meticulously regulated through intestinal absorption, bone storage, and kidney excretion to maintain serum levels within the narrow range of 2.2–2.5 mmol/L (Peacock, 2010). Active transcellular calcium transport across intestinal and renal epithelial barriers involves three steps: calcium enters epithelial cells through TRPV5 or TRPV6 channels, is transported intracellularly by calbindin-D9k or calbindin-D28k, and is then expelled across the basolateral membrane via Sodium-Calcium exchanger 1(NCX1) or the Ca^2+^-ATPase (PMCA1b) (van Goor et al., 2017).

TRPV5 and TRPV6 are recognized as calcium gatekeepers, playing essential roles in maintaining calcium balance under physiological conditions (van Goor et al., 2017). However, in the context of CKD-MBD, factors such as hypocalcemia, vitamin D deficiency, dysregulation of the FGF23-Klotho axis, as well as other internal environmental disturbances, can lead to dysfunction of these channels. Vitamin D exerts its effects by activating the vitamin D receptor (VDR), which subsequently dimerizes with the retinoic acid X receptor (RXR) to form a functional complex (Christakos et al., 2016). This VDR-RXR complex then activates vitamin D response elements (VDREs) located in the promoter regions of TRPV5 and TRPV6, thereby regulating their transcription and expression (Fleet, 2022). Mutations within these VDREs can render TRPV6 unresponsive to vitamin D. The decreased activity of 1α-hydroxylase leads to 1,25(OH)_2_D_3_ deficiency in the kidney of patients with CKD-MBD, which in turn leads to the decreased expression of TRPV5/TRPV6. At the same time, uremic toxins such as indoxyl sulfate inhibit VDR binding to target genes and aggravate calcium absorption disorders.

Additionally, parathyroid hormone stimulates the cAMP-protein kinase A (PKA) signaling pathway via adenylyl cyclase (ADCY), which leads to the rapid phosphorylation of threonine-709 on TRPV5, thereby enhancing calcium reabsorption in the distal nephron. This mechanism serves to prevent calcimimetics -induced inactivation of TRPV5 (Fluck et al., 2022). However, in the advanced stages of CKD-MBD, renal injury, deficiency of active vitamin D, and imbalance in the FGF23-Klotho axis result in the downregulation of TRPV5 and TRPV6. This dysregulation impairs the normal modulation of TRPV5 and TRPV6, subsequently promoting the secretion of PTH. In the initial stages of CKD, the body compensates by elevating levels of FGF23 and parathyroid hormone (PTH) to maintain serum phosphorus concentrations within the normal range. As the glomerular filtration rate further declines, FGF23 levels increase substantially, potentially up to 1,000-fold. Fibroblast growth factor 23 (FGF23) interacts with the FGFR1/Klotho complex to downregulate Type IIa Na^+^/Pi Cotransporter (Na/Pi-Ⅱa) and Na/Pi-Ⅱc transporters in the renal system and Na/Pi-Ⅱb transporters in the gastrointestinal tract (Erben and Andrukhova, 2015). However, the deficiency of Klotho impairs the phosphate excretion function of FGF23, leading to phosphorus retention within the body (Muñoz-Castañeda et al., 2020). FGF23 plays a crucial role in modulating the expression of vitamin D metabolism enzymes, specifically Cyp27b1 and Cyp24a1, thereby expediting the inactivation of 1,25-dihydroxyvitamin D. Additionally, FGF23 influences the translocation of TRPV5 from the Golgi apparatus to the plasma membrane, enhancing its surface expression through the activation of the ERK1/2-SGK1-WNK4 signaling pathway (Andrukhova et al., 2014). Elevated FGF23 is linked to negative outcomes like left ventricular hypertrophy and cardiovascular events.

**FIGURE 2:**
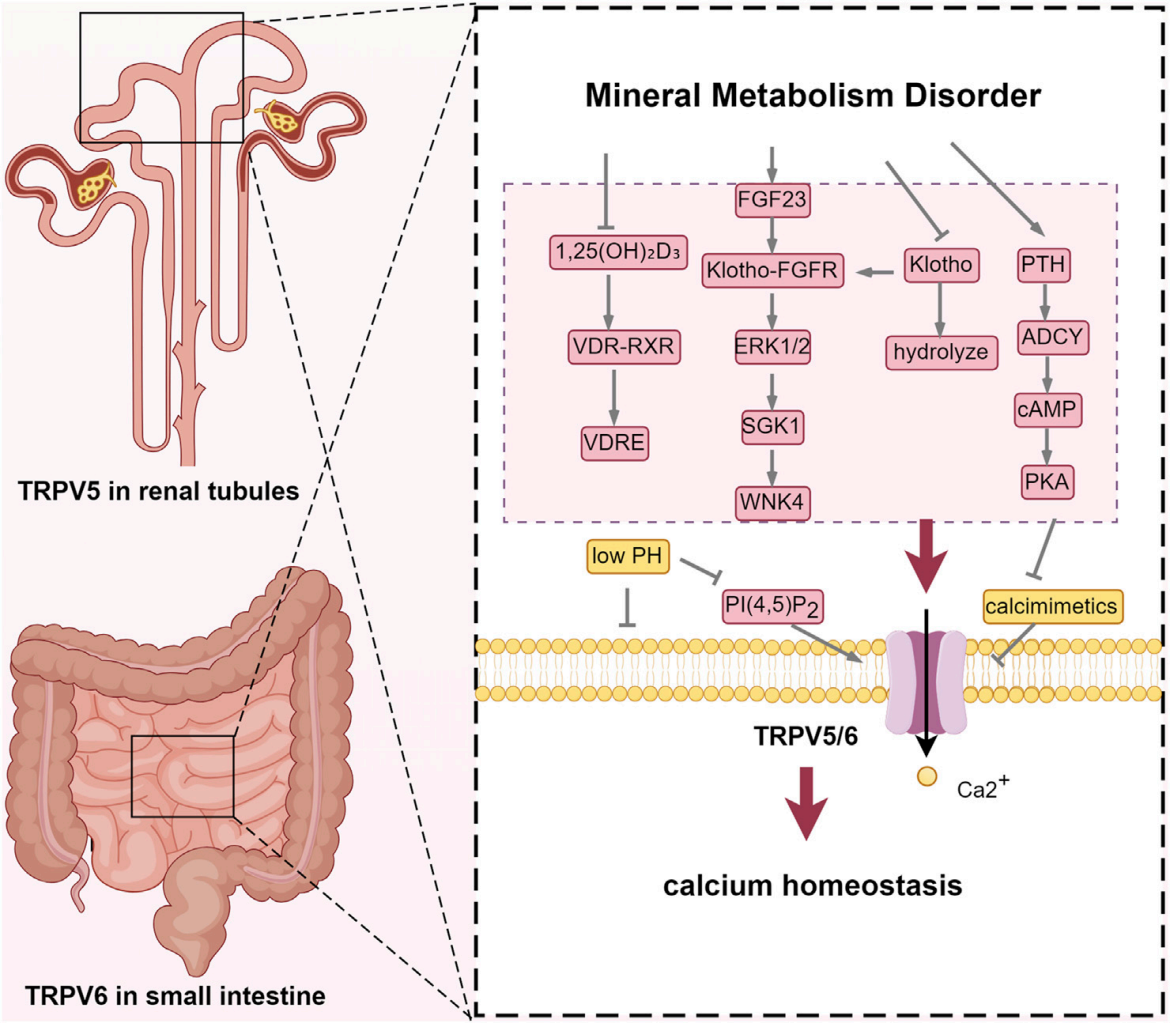
TRPV5 and TRPV6: regulators of calcium homeostasis. Abbreviations: VDR, vitamin D receptor; RXR, retinoic acid X receptor; VDRE, vitamin D response element; FGF23, fibroblast growth factor 23; FGFR: fibroblast growth factor receptor; ERK1/2, extracellular signal-regulated kinase 1/2; SGK1, Serum/Glucocorticoid Regulated Kinase 1; WNK4, WNK Lysine Deficient Protein Kinase 4; PTH, parathyroid hormone; ADCY, Adenylate Cyclase; cAMP, cyclic adenosine monophosphate; PKA, protein kinase A; PI(4,5)P_2_, phosphatidylinositol 4,5-bisphosphate.

Klotho protein exists in two forms, both of which enhance TRPV5 presence on the cell surface (Wolf et al., 2014). As an FGF23 co-receptor, membrane klotho regulates calcium and phosphorus metabolism by binding to FGFR (Kuro-o, 2010). Soluble klotho stabilizes TRPV5 on the membrane by cleaving terminal sialic acids, thereby exposing the N-acetylgalactosamine (GalNAc) residue to facilitate binding with galectin-1 (Huang, 2012; Leunissen et al., 2013; Dalton et al., 2017). Klotho also regulates the TRPV6 channel via N-glycosylation but does not affect TRPV4 and TRPM6 in the kidney, indicating TRPV5 and TRPV6’s specificity in calcium homeostasis regulation (Lu et al., 2008). Recent studies propose that soluble Klotho anchors TRPV5 to the membrane by directly binding to TRPV5 and galectin-1, warranting further investigation (Lee et al., 2021) (Figure 2).

A growing body of animal experimental studies has demonstrated that the dysfunction of TRPV5 and TRPV6 can disrupt calcium homeostasis (Bianco et al., 2007; Loh et al., 2013). Hoenderop et al. found that TRPV5 knockout mice exhibit severe hypercalciuria. *In vivo* micropuncture experiments revealed that Ca^2+^ reabsorption was impaired in DCT and CNT, which is consistent with the localization of TRPV5. Serum calcium levels did not decrease in these mice, as serum 1,25-(OH)_2_D_3_ levels were compensated by an upregulation of intestinal TRPV6 and calbindin-D_9K_ expression. Furthermore, trabecular and cortical bone thickness was significantly reduced in these mice (Hoenderop et al., 2003). It is concluded that the dysfunction of TRPV5 and TRPV6, leading to disturbances in calcium homeostasis and bone metabolism, contributes to the pathophysiological progression of CKD-MBD. Several studies have been consistent with the hypothesis that TRPV6 mediates calcium entry into the apical membrane of intestinal cells (Song et al., 2003; Khattar et al., 2022). TRPV6 knockout (KO) mice show a 60% reduction in intestinal calcium absorption, increased urinary calcium excretion, and decreased bone density (Bianco et al., 2007). However, a few studies have indicated that TRPV6 KO mice can still increase calcium absorption efficiency and maintain normal serum calcium levels under a low-calcium diet and 1,25(OH)_2_D injection (Benn et al., 2008). This seems to challenge the core role of TRPV6 in intestinal calcium absorption. Cui filled in the last piece of the puzzle, demonstrating that even in VDR KO mice, TRPV6 transgenic expression can reverse hypocalcemia and osteomalacia (Cui et al., 2012).

Hypercalcemia is becoming more common in CKD-MBD patients, and the death risk rises 3.49 times when serum calcium exceeds 10.9–11.9 mg/dL (Zhu et al., 2018). Fortunately, the regulation of calcium absorption by TRPV6 is not a one-way street. Elevated serum calcium concentrations or the administration of exogenous calcimimetic agents can suppress calcium absorption via TRPV6 by activating the calcium-sensing receptor (CaSR) in intestinal epithelial cells, thus mitigating the onset of hypercalcemia (Lee et al., 2019; Hou et al., 2022). This evidence suggests that calcimimetics may be more appropriate than vitamin D for patients with CKD-MBD who are experiencing hypercalcemia.

## 3 Dysregulated bone turnover: TRP channels and renal osteodystrophy

### 3.1 Renal osteodystrophy

According to KDIGO guidelines, renal bone disease is categorized based on three key parameters: bone turnover, bone mineralization, and bone mass. These categories include high-turnover bone diseases, such as osteitis fibrosa cystica; low-turnover bone diseases, such as adynamic bone disease and osteomalacia; and mixed bone diseases. Bone turnover encompasses two distinct processes: osteoclasts release acidic substances and proteases to dissolve minerals and degrade the bone matrix, thereby facilitating bone resorption, while osteoblasts synthesize and deposit osteoid to promote the formation and mineralization of new bone. The assessment of bone turnover is essential for determining the most suitable treatment for ROD (Khairallah and Nickolas, 2018). Bone undergoes continuous formation and resorption in response to mechanical loading, fluctuations in serum calcium levels, and various paracrine and endocrine factors. Consequently, modulating the channel activity of the highly Ca^2+^ selective TRPV5/TRPV6 and the mechanosensitive TRPV4 channels may influence the proliferation and differentiation of osteoblasts and osteoclasts.

### 3.2 TRPV5/6 and renal osteodystrophy: beyond calcium

As mentioned earlier, mice lacking TRPV5 exhibited reduced femoral cortical and trabecular bone thickness. Another animal experiment showed that TRPV5 was present in the wrinkling zone of osteoclasts. TRPV5 knockout mice showed increased osteoclast number and osteoclast area, but decreased urinary deoxypyridinoline, a marker of bone resorption, and inconspicuous bone resorption lacunans in osteoclasts cultured *in vitro* (van der Eerden et al., 2005). This seemingly paradoxical observation is due to the fact that deficiency of trpv5 leads to dysfunctional osteoclast production. Subsequent studies confirmed that, the persistent challenge of Ca^2+^ homeostasis in Trpv5−/− mice leads to faster bone aging and reduced cortical and trabecular bone mass (van der Eerden et al., 2016).

Bone turnover is modulated by the RANKL/RANK/OPG system. RANKL, secreted by osteoblasts, binds to the RANK receptor on osteoclast precursors, thereby facilitating their differentiation. Osteoprotegerin (OPG) functions as a decoy receptor, inhibiting this process by preventing RANKL from interacting with RANK. TRPV5/6 is involved in maintaining or amplifying RANKL-induced calcium oscillations, which make calcineurin more easily activated to dephosphorylate nuclear factor of activated T cells 1(NFATc1). Activated NFATc1 translocalizes to the nucleus and drives the expression of osteoclast differentiation genes. Inhibition of TRPV5 expression significantly diminishes the RANKL-induced rise in cytosolic Ca^2+^ and enhances bone resorption capacity (Chamoux et al., 2010), indicating that TRPV5 plays a role in maintaining human bone homeostasis via a negative feedback loop. Trpv6 suppressed osteoclastogenesis by decreasing the ratios of phosphoprotein/total protein in the IGF-PI3K-AKT signalling pathway (Ma et al., 2021). In TRPV6 KO mice, there is a significant reduction in bone mineral density and trabecular bone number (Chen et al., 2014).

Interestingly, van der Eerden et al. did not detect TRPV5 in osteoblasts; however, they identified tcalbindin-D_28k_, NCX1, and PMCA1b. It is plausible to speculate that the expression of TRPV5 channels necessitates specific conditional activation. Conversely, the presence of TRPV6 suggests that it may influence the functional activity of osteoblasts (Little et al., 2011).

### 3.3 TRPV4: linking mechanical stress to bone metabolism

**FIGURE 3:**
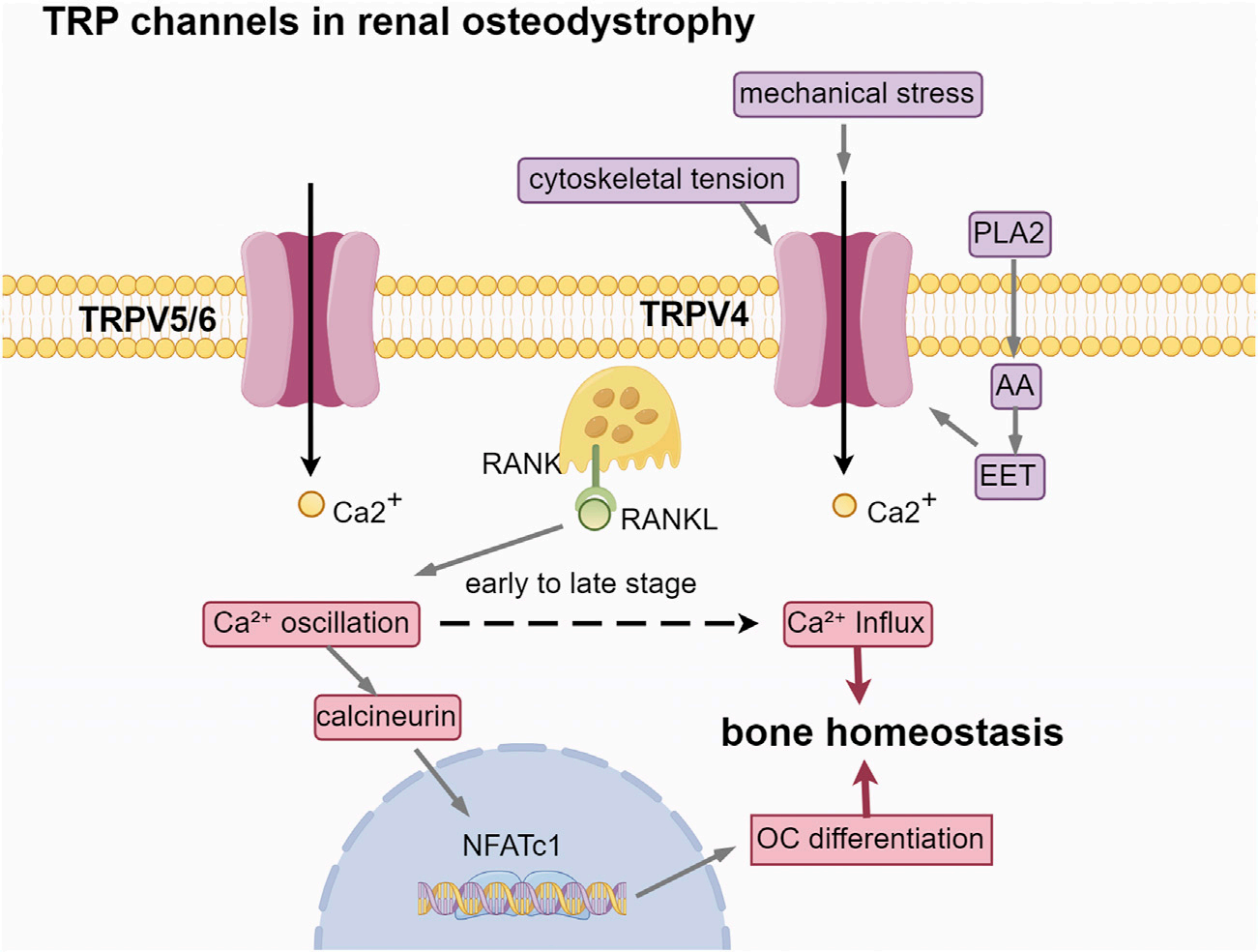
TRP channels in renal osteodystrophy. Abbreviations: PLA2, phospholipase A2; AA, arachidonic acid; EET, epoxyeicosatrienoic acids. RANKL, receptor activator of nuclear factor-κB ligand; RANK, receptor activator of nuclear factor-κB; NFATc1, nuclear factor of activated T cells 1; OC, osteoclast.

Another member of the TRPV family, the TRPV4 channel, is a non-selective cation channel characterized by its osmotic and mechanical sensitivity. The moderate permeability of TRPV4 to Ca^2+^ ions has been associated with the aspartate residues at positions 672 and 682. This channel is activated by stimuli such as heat, hypotonic conditions, mechanical forces, and various chemical agonists, including endocannabinoids, arachidonic acid metabolites, and phorbol esters. TRPV4 is widely expressed across several tissues, including the trachea, kidney, liver, lung, spleen, and skin. In bone tissue, TRPV4 is predominantly expressed on the surfaces of osteoclasts and osteoblasts (Everaerts et al., 2010). TRPV4, located on the primary cilia of osteocytes, plays a crucial role in detecting mechanical stress and osmotic pressure, subsequently transducing these mechanical stimuli into biochemical signals (Cox et al., 2019; Hruska and Mahjoub, 2019). Several studies indicate that fluid shear stress, resulting from the stretching of the cell membrane or the flow of interstitial fluid within the bone cavity, may directly modify the conformation of TRPV4, thereby facilitating the opening of this channel (Michalick and Kuebler, 2020). Alterations in cytoskeletal tension induced by cellular stretching, including the reorganization of actin, have the potential to modulate TRPV4 activity (Matthews et al., 2010). Mechanical stress may also indirectly activate TRPV4 through the activation of phospholipase A2 (PLA2), leading to the release of arachidonic acid (AA) and its metabolites, including epoxyeicosatrienoic acids (EETs) (Berna-Erro et al., 2017). In mature osteoclasts, TRPV4 is specifically localized to the basolateral membrane, where it plays a regulatory role in Ca^2+^ influx (Li et al., 2018). During the late stages of osteoclast differentiation, there is a reduction in calcium oscillations, while TRPV4-mediated basolateral calcium influx becomes more prominent, gradually supplanting calcium oscillations as the primary intracellular calcium signaling mechanism (Masuyama et al., 2008). Lauren Hurd isolated primary chondrocytes from patients with TRPV4 mutations, discovering an increased peak amplitude of temperature-dependent oscillations [(Ca^2+^)i] (Hurd et al., 2015). This finding suggests that aberrant activation of TRPV4 contributes to severe cartilage dysplasia. Numerous *in vitro* studies examining the function of TRPV4 through cell transfection (Camacho et al., 2010; Loukin et al., 2011). In animal models, the deficiency of TRPV4 led to an increase in bone mass in mice, primarily attributable to the compromised function of osteoclast-mediated bone resorption. Furthermore, the deletion of TRPV4 prevented the typical reduction in bone formation and the enhancement of bone resorption associated with mechanical unloading (Mizoguchi et al., 2008). This underscores the significance of TRPV4 as a crucial target for mechanosensitive channels in the maintenance of bone homeostasis (Figure 3).

## 4 Ectopic calcium deposition: TRP channels and vascular calcification

### 4.1 Vascular calcification

**FIGURE 4:**
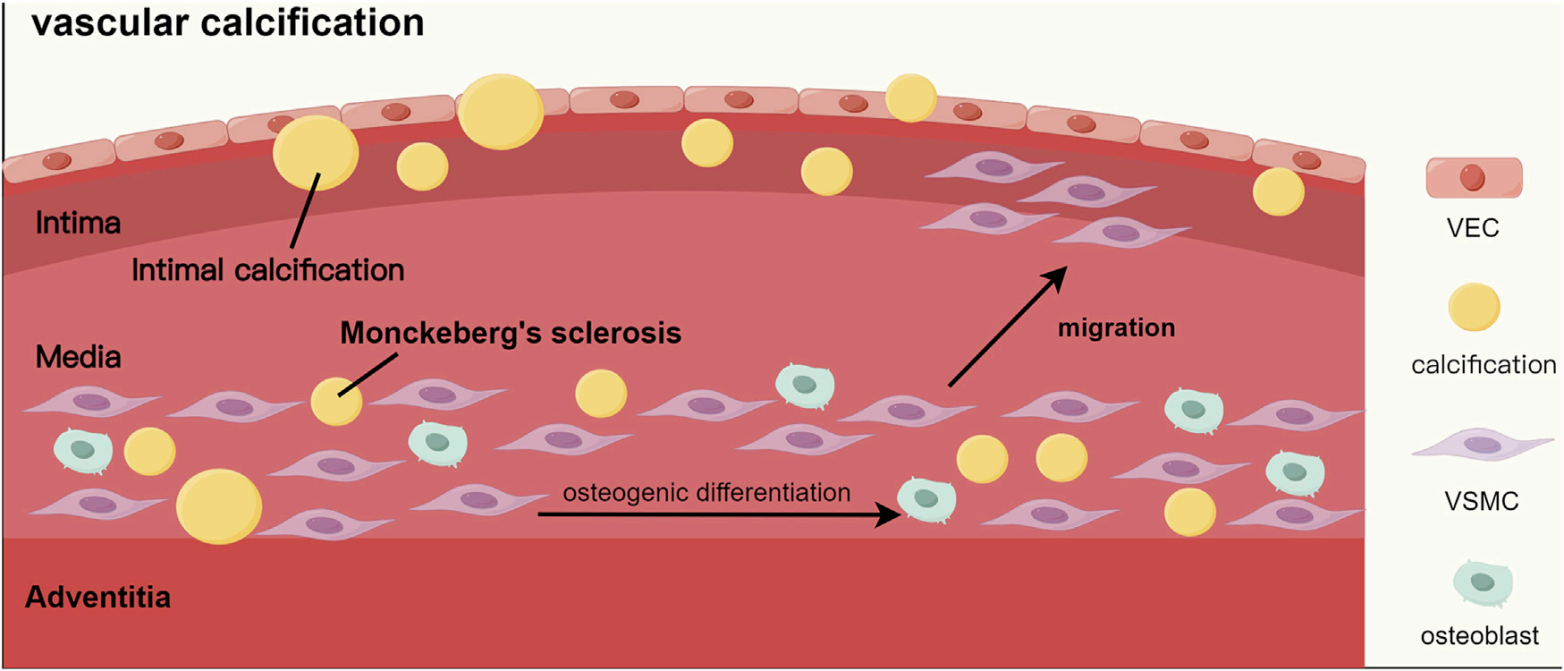
The process of vascular calcification. Abbreviations: VEC, vascular endothelial cell; VSMC, vascular smooth muscle cell.

Vascular calcification, a key risk factor for cardiovascular events in CKD-MBD patients, has two types: intimal calcification and medial calcification. Intimal calcification affects the aorta, coronary, and carotid arteries. It is characterized by calcification located at the interface between the lipid necrotic core and the fibrous cap, leading to unstable plaques and thrombosis risk. Medial calcification, known as Monckeberg’s sclerosis, occurs in peripheral arteries like the femoral and tibial (Rogers et al., 2013). Both types result from vascular smooth muscle cells transforming into osteoblast-like cells, marked by the expression of osteogenic markers like Runx2 and osteocalcin, as well as regulatory proteins such as BMP-2 and ALP, rather than merely the passive deposition of calcium and phosphorus (Kaur and Singh, 2022) (Figure 4).

Furthermore, patients with end-stage renal disease (ESRD) frequently present with multisystem calcified lesions. Cardiac valve calcification (CVC) is characterized by the calcification of the aortic valve and mitral annulus (Bai et al., 2022; Wang et al., 2023). Additionally, calciphylaxis is pathologically characterized by the calcific occlusion of subcutaneous microvessels, leading to thrombotic skin necrosis (Gallo Marin et al., 2023).

### 4.2 TRPC3: mediator of inflammatory signaling in vascular calcification

TRPC3 is a member of the TRPC family, which is frequently regarded as a store-operated calcium channel (Bavencoffe et al., 2017). TRPC3 shares a high degree of homology with TRPC6, and both are activated by diacylglycerol. TRPC proteins are broadly expressed in vascular endothelial cells and smooth muscle cells. TRPC3 and TRPC6 are primarily found in prerenal resistance vessels, while TRPC1 and TRPC6 are mainly in the aorta. TRPC regulates smooth muscle activity, vascular permeability, and is linked to cardiac hypertrophy and endothelial dysfunction (Wen et al., 2020).

**FIGURE 5:**
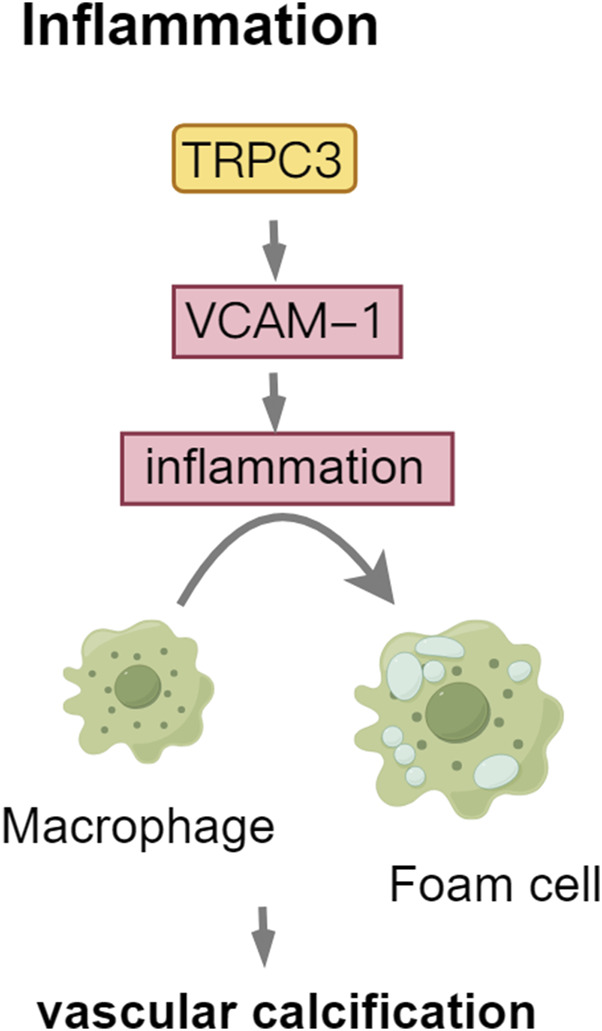
TRPC3: mediator of Inflammatory Signaling in vascular calcification. Abbreviations: VCAM-1, vascular cell adhesion molecule-1.

TRPC3 and TRPC6 are implicated in inflammatory and immune processes, including macrophage activation (Parenti et al., 2016). CKD is characterized by persistent low-grade inflammation. Cells orchestrate the immune response by expressing and releasing inflammatory mediators, which recruit additional inflammatory cells, thereby amplifying the inflammatory response and exacerbating the injury (Ebert et al., 2020). Intimal calcification results from inflammation, linked to inflammatory molecules expressed by endothelial and vascular smooth muscle cells, which encourage macrophage infiltration. *In vitro* and *in vivo* studies have demonstrated that TRPC3 overexpression enhances vascular cell adhesion molecule-1(VCAM-1) expression, promoting endothelial cell inflammation and exacerbating the progression of atherosclerotic lesions through increased calcium influx (Smedlund et al., 2010; 2015; Vazquez et al., 2016). Inflammatory mediators facilitate the recruitment of monocytes into the subintimal space, where they differentiate into foam cells, thereby contributing to plaque development and increasing the risk of plaque rupture (Martini et al., 2023). TRPC3 encourages macrophages to polarize into the M1 type, which releases pro-inflammatory cytokines (IL-1β, TNF-α, IL-6) that trigger osteogenic differentiation and apoptosis in VSMCs. The depletion of TRPC3 significantly reduces plaque calcification and the expression of osteogenesis-related genes, such as Runx-2, Bmp-2, and ALP, particularly in macrophage-rich regions (Kumarasamy et al., 2017; Solanki et al., 2017; Dube et al., 2018). These findings underscore the critical role of TRPC3 in facilitating BMP-2-dependent calcification processes in macrophages (Figure 5).

### 4.3 TRPM2: enhancing oxidative stress in vascular calcification

The TRPM subfamily comprises eight members, designated as TRPM1 through TRPM8. TRPM channels are implicated in various pathological processes associated with cardiovascular diseases (Hiroi et al., 2013; Mittal et al., 2015). TRPM2, a non-selective cation channel within the TRPM subfamily, is extensively expressed in the brain, bone marrow, spleen, heart, lungs, immune cells, and vascular endothelium. This channel facilitates the influx of Na^+^ and Ca^2+^ ions and can be activated by oxidative stress induced by ROS (Di et al., 2011), thereby increasing cellular susceptibility to apoptosis. In the context of CKD, cellular oxygen metabolism becomes dysregulated, impairing mitochondrial capacity to eliminate oxygen free radicals. This impairment results in the accumulation of ROS, thereby exacerbating renal damage progression, particularly through lipid oxidation. In VSMCs, oxidative stress modulates RUNX2 via the PI3K/AKT signaling pathway.

**FIGURE 6:**
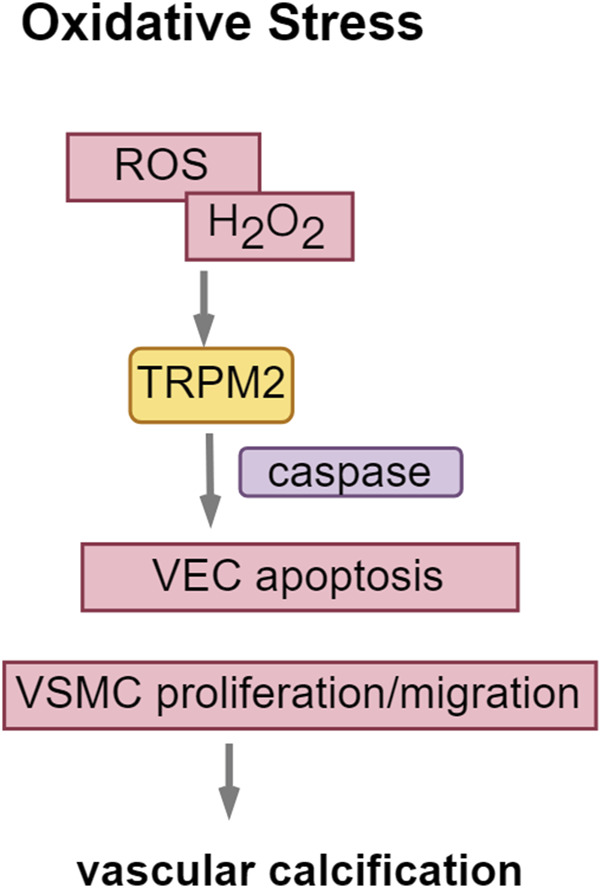
TRPM2: enhancing oxidative stress in vascular calcification. Abbreviations: ROS, reactive oxygen species; H_2_O_2_, hydrogen peroxide.

ROS activate the PARP1-dependent TRPM2 receptor, contributing to vascular dysfunction (Alves-Lopes et al., 2020). Hydrogen peroxide (H_2_O_2_)-induced activation of TRPM2 facilitates the proliferation and migration of aortic vascular smooth muscle cells via the Akt signaling pathway and contributes to neointimal hyperplasia within the vascular wall (Zhou et al., 2020). This process serves as a marker for intimal calcification. Furthermore, the overexpression of TRPM2 exacerbates hydrogen peroxide (H_2_O_2_)-induced apoptosis in vascular endothelial cells, a process linked to the activation of caspase-8, caspase-9, and caspase-3 (Hecquet et al., 2010; Sun et al., 2012) (Figure 6).

### 4.4 TRPM7: a double-edged sword in vascular calcification

TRPM7 is a divalent cation channel that facilitates the permeation of calcium and magnesium ions, playing a crucial role in the regulation of magnesium ion homeostasis (Zou et al., 2019). Additionally, TRPM7 possesses serine/threonine protein kinase activity. It is ubiquitously expressed throughout the human body, with particularly high expression levels in the intestine, liver, nervous system, kidney, and cardiac (Fleig and Chubanov, 2014; Abidin et al., 2025).

Calcification transpires when the calcium-phosphorus product, alkaline phosphatase (ALP), RUNX2, and other promoting factors surpass the inhibitory effects of calcification inhibitors, including Gla protein, alpha-fetoprotein, and bone morphogenetic protein-7. Magnesium inhibits vascular calcification by boosting anti-calcification proteins like osteopontin, BMP-7, and matrix Gla protein, while also blocking the Wnt/β-catenin signaling pathway (Montezano et al., 2010; Montes de Oca et al., 2014). TRPM7 is recognized for its role in regulating transmembrane magnesium transport and is expressed in VSMCs. However, the role of TRPM7 in vascular calcification is complex. In cellular experiments, Mg^2+^ entry through TRPM7 channels significantly reduced VSMC calcification, as shown by the attenuation of this effect by 2-APB, a TRPM7 inhibitor. This inhibition byMg^2+^ was not achieved by calcium substitution or excess accumulation (Louvet et al., 2013; Sonou et al., 2017). In the CKD model, calcitriol treatment lowered TRPM7 protein levels in blood vessels, whereas combining magnesium with calcitriol reduced vascular calcification by boosting TRPM7 expression and magnesium levels in the vascular microenvironment (Zelt et al., 2015). However, it has also been documented that Mg^2+^ appear to exert their anti-calcification effects primarily through the inhibition of hydroxyapatite crystal formation, rather than through the modulation of TRPM7 channels (Ter Braake et al., 2018). In ESRD patients, uremic toxin indoxyl sulfate (IS) builds up, while high phosphorus converts vascular smooth muscle cells into osteoblast-like cells through a sodium-phosphate exchanger. Both are high risk factors for vascular calcification. In the cellular experiment, phosphate strongly promoted vascular endothelial cell calcification and increased TRPM7 levels, whereas IS caused weaker calcification and reduced TRPM7 expression (Lee et al., 2020). TRPM7 had opposite effects on calcification induced by phosphate and IS, indicating its complex role in vascular calcification.

**FIGURE 7:**
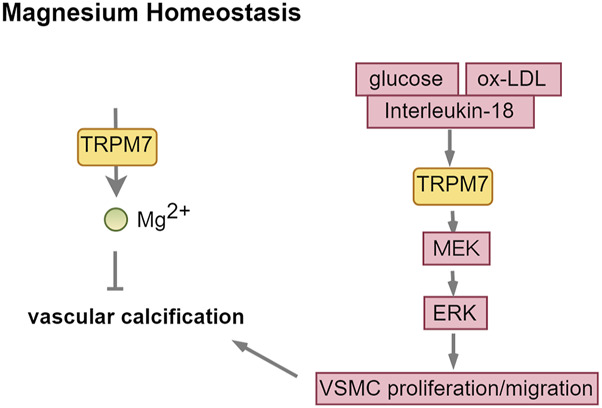
TRPM7: a double-edged sword in vascular calcification. Abbreviations: ox-LDL, oxidized low-density lipoprotein; MEK, MAPK/ERK Kinase; ERK, extracellular regulated protein kinases.

TRPM7 plays a crucial role in key cellular processes like proliferation, differentiation, adhesion, migration, and apoptosis. Elevated glucose levels or oxidized low-density lipoprotein (ox-LDL) activates TRPM7. This activation of TRPM7 enhances the proliferation, migration, and phenotypic transformation of vascular smooth muscle cells via the MEK-ERK signaling pathway, consequently advancing the progression of vascular calcification (Lin et al., 2016; Yang et al., 2017). Interleukin-18 enhances VSMC osteogenic differentiation and calcification via the ERK1/2 pathway (Zhang et al., 2017), indicating TRPM7 might link inflammation to vascular calcification (Figure 7; Table 1).

**TABLE 1:** The functions of TRP channels in CKD-MBD.

Channels	Distributions	Targets	Functions	References
TRPV5	Kidney, small intestine, pancreas, testis, prostate, placenta, brain, colon, rectum	Osteoclast, apical plasma membrane of DCT2 and CNT epithelial cells	Participating in RANKL-induced calcium oscillations → regulating early proliferation and differentiation of osteoclasts → negative feedback maintaining bone homeostasis	Chamoux et al. (2010) and van der Eerden et al. (2016)
TRPV6	Gastrointestinal tract (esophagus, stomach, duodenum, jejunum, ileum, colon), pancreas, placenta, prostate, salivary gland, liver, kidney, testis	Brush border of small intestinal epithelial cells, osteoblast, osteoclast	High calcium selectivity, mediating renal/intestinal calcium reabsorption/absorption → bidirectionally regulating serum calcium levels, preventing hypercalciuria → maintaining calcium homeostasis	van Goor et al. (2017) and Diver et al. (2022)
			Pathological conditions including acidosis, vitamin D deficiency, SHPT, and FGF23-Klotho axis disorder → functional abnormalities → exacerbating calcium metabolic disorder	Maeda et al. (2013), Andrukhova et al. (2014), Fleet, 2022, and Fluck et al. (2022)
			Regulating osteoclast *via* IGF-PI3K-AKT and other signaling pathways without involving calcium oscillations → maintaining bone homeostasis	Chen et al. (2014) and Ma et al. (2021)
TRPV4	Kidney, intestine, nervous system, muscle, lung, skin, pancreas, blood, liver, adrenal gland, eye, bone	Osteoblast, osteoclast, chondrocte	Mechanosensitivity → Converting mechanical stimuli into biochemical signals	Michalick and Kuebler (2020), Matthews et al. (2010), Berna-Erro et al. (2017), and Mizoguchi et al. (2008)
			Mediating calcium influx replacing calcium oscillations → Regulating late-stage proliferation and differentiation of osteoclast → Maintaining bone homeostasis	Masuyama et al. (2008) and Li et al. (2018)
			Maintaining normal development and differentiation of cartilage	Hurd et al. (2015)
TRPC3	Nervous-system, Muscle, Blood, Heart, Kidney, Lung, Pancreas	VEC, pericytes, macrophages	Participating in macrophage activation/promoting endothelial cell adhesion molecule expression → mediating inflammatory responses → promoting vascular calcification progression	Smedlund et al. (2010), Smedlund et al. (2015), Vazquez et al. (2016)
				Kumarasamy et al. (2017), Solanki et al. (2017), and Dube et al. (2018)
TRPM2	Nervous system, bone marrow, heart, pancreas, muscle, blood, lung	VEC, VSMC, macrophages and other vascular-related immune cells	Participating in oxidative stress and inflammatory responses → promoting proliferation, migration of VSMCs and vascular dysfunction/promoting apoptosis of VECs→ accelerating ascular calcification progression	Alves-Lopes et al. (2020), Hecquet et al. (2010), and Sun et al. (2012)
TRPM7	Intestine, liver, nervous system, kidney, heart, muscle, lung, pancreas, spleen, skin, lymph node, blood, adrenal gland, stomach, bone marrow, thyroid gland, bone	VEC, VSMC, fibroblast macrophages	Mediating magnesium ion influx → Participating in magnesium-dependent inhibition of VSMC calcification	Louvet et al. (2013), Zelt et al. (2015), and Sonou et al. (2017)
			Participating in cell proliferation, differentiation, adhesion, migration, and apoptosis → Promoting VSMC proliferation, migration, and phenotypic switching via the MEK-ERK pathway	Lin et al. (2016) and Yang et al. (2017)
			Integrating inflammatory signaling with vascular calcification cascades	Zhang et al. (2017)

Abbreviations: DCT2, distal convoluted tubule; CNT, connecting tubule; RANKL, receptor activator of nuclear factor-κB ligand; SHPT, secondary hyperparathyroidism; FGF23, fibroblast growth factor 23; VEC, vascular endothelial cell; VSMC, vascular smooth muscle cells.

## 5 Therapeutic frontiers: targeting TRP channels in CKD-MBD

At present, no pharmacological agents targeting TRP channels have received approval for the treatment of CKD-MBD. Nonetheless, numerous studies, including both clinical and basic research trials, are investigating TRP channels as potential therapeutic targets. Given that TRP channels are primarily recognized for their roles as sensors of temperature, pain, and touch, a significant body of research has concentrated on CKD-MBD-induced bone pain and skin pruritus. Bone pain is associated with disrupted mineral metabolism, whereas pruritus is more closely linked to the accumulation of uremic toxins, inflammation, and neuropathy. Both symptoms contribute to sleep disturbances, depression, and anxiety, thereby adversely impacting patients’ quality of life.

Capsaicin, as a recognized agonist of TRPV1, can inhibit the Wnt/β-catenin signaling pathway by activating TRPV1 receptor, thereby reducing the expression of RUNX2 and BMP-2 genes and helping to reduce arterial vascular calcification (Yan et al., 2024). In addition, it has been found that capsaicin can upregulate the expression of SIRT6, which leads to the deacetylation and degradation of Hif1α and prevents arterial calcification (Luo et al., 2022). GSK1016790A, a potent TRPV4 agonist, exerts chondroprotective effects on interleukin-1β-stimulated articular cartilage by activating the CaMKK/AMPK signaling pathway and inhibiting the NF-κB pathway (Hattori et al., 2021). In animal studies, 4α-PDD, a selective TRPV4 agonist, was shown to enhance calcium signaling and maintain osteoblast stability. Conversely, GSK205, a specific TRPV4 antagonist, was found to inhibit intracellular calcium oscillations and associated responses (Ozcivici et al., 2010). Additionally, anandamide and arachidonic acid have been demonstrated to activate TRPV4 channels, thereby increasing calcium influx and promoting vasodilation. Oxoglaucine, a TRPV5 inhibitor, has demonstrated efficacy in preventing cartilage degeneration by obstructing TRPV5-mediated Ca^2+^ active transmembrane transport and inducing autophagy (Zhong et al., 2021). Similarly, Alendronate, another TRPV5 inhibitor, has been shown to enhance bone mineral density and quality in patients with osteoporosis through the inhibition of TRPV5-mediated Ca^2+^ transmembrane transport (Nijenhuis et al., 2008). In animal models, TRPV5 blockers such as ML204 and AC1903 have exhibited renoprotective properties, including the prevention of podocyte loss and proteinuria, as well as the delay of renal function decline. Additionally, inhibitors targeting α-Klotho enzyme activity may serve as regulators of TRPV5 channels (Maeda et al., 2013). N-(p-aminocinnamoyl) anthranilic acid (ACA), a TRPM2 antagonist, has been demonstrated to inhibit the development of atherosclerosis in murine models. Additionally, ACA was observed to downregulate the expression of inflammatory mediators, including ICAM-1, MCP-1, and TNFα (Zhang et al., 2022). Furthermore, 2-APB, a non-selective TRPM7 inhibitor, has been shown to attenuate magnesium’s inhibitory effect on the calcification of smooth muscle cells *in vitro* (Louvet et al., 2013).

Simultaneously, certain traditional Chinese medicines exhibit potential therapeutic effects on CKD-MBD. Fraxinellone, a constituent of Dictamnus dasycarpus root bark, demonstrates anti-inflammatory and antipruritic properties through the modulation of TRPV1 and TRPA1 protein levels (Yang et al., 2024). Sophocarpine, an active compound in Flahorae Flavesentis Aiton, has been shown in studies to exert anti-inflammatory, antipruritic, and analgesic effects by modulating TRPA1 and TRPV1 (Zeng et al., 2025). Additionally, traditional Chinese medicine formulations, such as Shaoyao Gancao decoction, Qisheng Wan decoction, and Yu-Xue-Bi tablets, have been observed to regulate TRP channels, thereby alleviating bone and nerve pain (Luo et al., 2023; Liu et al., 2024; Wei et al., 2025). Scutellarein has been found to reduce pruritus in dermatitis patients by downregulating TRPV3 channels (Wang et al., 2022). Clinical trials indicate that moxibustion can alleviate pain through the regulation of TRPV1 channels (Jiang et al., 2016), while animal studies suggest that acupuncture can mitigate pain by modulating TRPV2 channels (Huang et al., 2018) (Table2).

**TABLE 2:** TRP channel-targeting drugs.

Channels	Therapeutic	Functions	Treatment effect	References
TRPV1	Capsaicin	Activate TRPV1 receptors	Reduce arterial vascular calcification, alleviate bone pain	Yan et al. (2024) and Luo et al. (2022)
		Inhibit the Wnt/β-catenin signaling pathway→reduce the expression of RUNX2 and BMP-2 genes→suppress arterial vascular calcification		
		Upregulating the expression of SIRT6→suppress arterial vascular calcification		
TRPV1	Moxibustion	Modulating the TRPV1 channel to alleviate pain	Relieve pain	Jiang et al. (2016)
TRPV2	Acupuncture	Modulating the TRPV2 channel to alleviate pain	Relieve pain	Huang et al. (2018)
TRPV1/TRPA1	Fraxinellone	Regulating the protein levels of TRPV1 and TRPA1, to exert anti-inflammatory and anti-itch effects	Alleviate itching and inflammation	Yang et al. (2024)
TRPA1/TRPV1	Sophocarpine	Modulating TRPA1 and TRPV1 to exert anti-inflammatory, anti-itch, and analgesic effects	Alleviate itching and pain	Zeng et al. (2025)
TRPV3	Scutellarein	Downregulating the TRPV3 channel alleviates itching in patients with eczema	Relieve itching	Wang et al. (2022)
TRPV4	GSK1016790A	Activate the CaMKK/AMPK signaling pathway, inhibit the NF-κB signaling pathway → exert a protective effect on cartilage	Protect joint cartilage, reduce inflammation	Hattori et al. (2021)
TRPV4	4α-PDD	Enhance calcium signaling, maintain osteoblast stability	Promote bone stability	Ozcivici et al. (2010)
TRPV4	GSK205	Inhibit intracellular calcium oscillations and related reactions	Inhibition of endothelial calcification	Ozcivici et al. (2010)
TRPV4	Anandamide, Arachidonic acid	Activate the TRPV4 channel, increase calcium influx, and promote vasodilation	Alleviate vascular spasm, improve blood flow	Zhong et al. (2021)
TRPV5	Oxoglaucine	Block TRPV5-mediated Ca^2+^ transmembrane transport and induce autophagy	Prevent cartilage degeneration	Zhong et al. (2021)
TRPV5	Alendronate	Inhibit TRPV5-mediated Ca^2+^ transmembrane transport	Enhance bone density and quality, treat osteoporosis	Nijenhuis et al. (2008)
TRPV5	ML204, AC1903	Block the TRPV5 channel to prevent podocyte loss and proteinuria	Delay the decline in renal function	Maeda et al. (2013)
TRPM2	ACA	Inhibit the development of atherosclerosis, downregulate the expression of inflammatory mediators	Slow down the progression of atherosclerosis	Zhang et al. (2022)
TRPM7	2-APB	Inhibit TRPM7→Reducing magnesium’s inhibition of VSMC calcification	Inhibit vascular calcification	Louvet et al. (2013)

The targeted regulation of TRP channels presents a promising therapeutic strategy for CKD-MBD management, with dual potential to alleviate clinical symptoms and establish novel treatment paradigms. However, given the ubiquitous distribution of TRP channels in human tissues, therapeutic development must prioritize organ-specific targeting to minimize systemic side effects from non-selective activation or inhibition. This biological characteristic underscores the critical need for developing tissue-selective drug delivery systems or channel modulators with spatial specificity. Concurrently, traditional Chinese medicine (TCM), with its historical efficacy, warrants exploration as a complementary therapy. Integrating precision TRP targeting with optimized TCM may synergistically enhance efficacy and safety.

## 6 Conclusion

This review systematically examines the involvement of TRP channels in CKD-MBD, encompassing molecular mechanisms and potential therapeutic developments. The discussion commences with calcium, a mineral essential for life yet perilous in excess. TRPV5 and TRPV6, channels with high selectivity for calcium, are identified as pivotal regulators of calcium homeostasis. Their dysfunction due to CKD-MBD worsens mineral disorders and renal bone disease. Additionally, the mechanosensitive TRPV4 channel acts as a transducer, converting mechanical stimuli into biochemical signals to maintain bone remodeling homeostasis. Vascular calcification and bone loss, although seemingly opposing processes, occur concurrently. TRPC3 and TRPM2 contribute to vascular calcification via inflammatory and oxidative stress pathways, whereas TRPM7 exhibits a paradoxical function: it is essential for maintaining magnesium homeostasis, yet its role in magnesium mediated anti-calcification remains an unresolved enigma.

Preclinical studies offer hope. Pharmacological agents targeting TRP channels, like channel blockers and allosteric modulators, hold promise in mitigating the phenotypic manifestations of CKD-MBD by decreasing vascular calcification and maintaining bone density, among other effects. Traditional Chinese medicine therapy is also beneficial. Nevertheless, key challenges remain: (1) Achieving selective activation of specific TRP channel subtypes without off-target effects due to their structural similarity; (2) Developing organ-specific targeting strategies to avoid systemic interactions given their widespread tissue distribution. The story of TRP channels and CKD-MBD is ongoing, requiring further experimental research and multicenter clinical trials to turn insights into clinical advances.

## References

[B1] Abidin B. M. Rios F. J. Montezano A. C. Touyz R. M. (2025). Transient receptor potential melastatin 7 cation channel, magnesium and cell metabolism in vascular health and disease. Acta Physiol. (Oxf) 241, e14282. 10.1111/apha.14282 39801180

[B2] Alves-Lopes R. Neves K. B. Anagnostopoulou A. Rios F. J. Lacchini S. Montezano A. C. (2020). Crosstalk between vascular redox and calcium signaling in hypertension involves TRPM2 (transient receptor potential melastatin 2) cation channel. Hypertension 75, 139–149. 10.1161/hypertensionaha.119.13861 31735084

[B3] Andrukhova O. Smorodchenko A. Egerbacher M. Streicher C. Zeitz U. Goetz R. (2014). FGF23 promotes renal calcium reabsorption through the TRPV5 channel. EMBO J. 33, 229–246. 10.1002/embj.201284188 24434184 PMC3983685

[B4] Bai J. Zhang X. Zhang A. Zhang Y. Ren K. Ren Z. (2022). Cardiac valve calcification is associated with mortality in hemodialysis patients: a retrospective cohort study. BMC Nephrol. 23, 43. 10.1186/s12882-022-02670-5 35065601 PMC8783521

[B5] Bavencoffe A. Zhu M. X. Tian J.-B. (2017). New aspects of the contribution of ER to SOCE regulation: TRPC proteins as a link between plasma membrane ion transport and intracellular Ca2+ stores. Adv. Exp. Med. Biol. 993, 239–255. 10.1007/978-3-319-57732-6_13 28900918

[B6] Benn B. S. Ajibade D. Porta A. Dhawan P. Hediger M. Peng J.-B. (2008). Active intestinal calcium transport in the absence of transient receptor potential vanilloid type 6 and calbindin-D9k. Endocrinology 149, 3196–3205. 10.1210/en.2007-1655 18325990 PMC2408805

[B7] Berna-Erro A. Izquierdo-Serra M. Sepúlveda R. V. Rubio-Moscardo F. Doñate-Macián P. Serra S. A. (2017). Structural determinants of 5’,6’-epoxyeicosatrienoic acid binding to and activation of TRPV4 channel. Sci. Rep. 7, 10522. 10.1038/s41598-017-11274-1 28874838 PMC5585255

[B8] Bianco S. D. C Peng J.-B. Takanaga H. Suzuki Y. Crescenzi A. Kos C. H. (2007). Marked disturbance of calcium homeostasis in mice with targeted disruption of the Trpv6 calcium channel gene. J. Bone Min. Res. 22, 274–285. 10.1359/jbmr.061110 PMC4548943 17129178

[B9] Bokhovchuk F. M. Bate N. Kovalevskaya N. V. Goult B. T. Spronk C. A. E. M. Vuister G. W. (2018). The structural basis of calcium-dependent inactivation of the transient receptor potential vanilloid 5 channel. Biochemistry 57, 2623–2635. 10.1021/acs.biochem.7b01287 29584409

[B10] Camacho N. Krakow D. Johnykutty S. Katzman P. J. Pepkowitz S. Vriens J. (2010). Dominant TRPV4 mutations in nonlethal and lethal metatropic dysplasia. Am. J. Med. Genet. A 152A, 1169–1177. 10.1002/ajmg.a.33392 20425821 PMC4169191

[B11] Chamoux E. Bisson M. Payet M. D. Roux S. (2010). TRPV-5 mediates a receptor activator of NF-kappaB (RANK) ligand-induced increase in cytosolic Ca2+ in human osteoclasts and down-regulates bone resorption. J. Biol. Chem. 285, 25354–25362. 10.1074/jbc.M109.075234 20547482 PMC2919098

[B12] Chen F. Ni B. Yang Y. O. Ye T. Chen A. (2014). Knockout of TRPV6 causes osteopenia in mice by increasing osteoclastic differentiation and activity. Cell Physiol. Biochem. 33, 796–809. 10.1159/000358653 24686448

[B13] Christakos S. Dhawan P. Verstuyf A. Verlinden L. Carmeliet G. (2016). Vitamin D: metabolism, molecular mechanism of action, and pleiotropic effects. Physiol. Rev. 96, 365–408. 10.1152/physrev.00014.2015 26681795 PMC4839493

[B14] Cox C. D. Bavi N. Martinac B. (2019). Biophysical principles of ion-channel-mediated mechanosensory transduction. Cell Rep. 29, 1–12. 10.1016/j.celrep.2019.08.075 31577940

[B15] Cui M. Li Q. Johnson R. Fleet J. C. (2012). Villin promoter-mediated transgenic expression of transient receptor potential cation channel, subfamily V, member 6 (TRPV6) increases intestinal calcium absorption in wild-type and vitamin D receptor knockout mice. J. Bone Min. Res. 27, 2097–2107. 10.1002/jbmr.1662 PMC3430830 22589201

[B16] Dalton G. D. Xie J. An S.-W. Huang C.-L. (2017). New insights into the mechanism of action of soluble klotho. Front. Endocrinol. (Lausanne) 8, 323. 10.3389/fendo.2017.00323 29250031 PMC5715364

[B17] Di A. Gao X.-P. Qian F. Kawamura T. Han J. Hecquet C. (2011). The redox-sensitive cation channel TRPM2 modulates phagocyte ROS production and inflammation. Nat. Immunol. 13, 29–34. 10.1038/ni.2171 22101731 PMC3242890

[B18] Diver M. M. Lin King J. V. Julius D. Cheng Y. (2022). Sensory TRP channels in three dimensions. Annu. Rev. Biochem. 91, 629–649. 10.1146/annurev-biochem-032620-105738 35287474 PMC9233036

[B19] Dube P. R. Chikkamenahalli L. L. Birnbaumer L. Vazquez G. (2018). Reduced calcification and osteogenic features in advanced atherosclerotic plaques of mice with macrophage-specific loss of TRPC3. Atherosclerosis 270, 199–204. 10.1016/j.atherosclerosis.2017.12.025 29290366 PMC5835414

[B20] Ebert T. Pawelzik S.-C. Witasp A. Arefin S. Hobson S. Kublickiene K. (2020). Inflammation and premature ageing in chronic kidney disease. Toxins (Basel) 12, 227. 10.3390/toxins12040227 32260373 PMC7232447

[B21] Erben R. G. Andrukhova O. (2015). FGF23 regulation of renal tubular solute transport. Curr. Opin. Nephrol. Hypertens. 24, 450–456. 10.1097/MNH.0000000000000145 26125643

[B22] Everaerts W. Nilius B. Owsianik G. (2010). The vanilloid transient receptor potential channel TRPV4: from structure to disease. Prog. Biophys. Mol. Biol. 103, 2–17. 10.1016/j.pbiomolbio.2009.10.002 19835908

[B23] Fleet J. C. (2022). Vitamin D-mediated regulation of intestinal calcium absorption. Nutrients 14, 3351. 10.3390/nu14163351 36014856 PMC9416674

[B24] Fleig A. Chubanov V. (2014). TRPM7. Handb. Exp. Pharmacol. 222, 521–546. 10.1007/978-3-642-54215-2_21 24756720 PMC5663634

[B25] Fluck E. C. Yazici A. T. Rohacs T. Moiseenkova-Bell V. Y. (2022). Structural basis of TRPV5 regulation by physiological and pathophysiological modulators. Cell Rep. 39, 110737. 10.1016/j.celrep.2022.110737 35476976 PMC9088182

[B26] Foreman K. J. Marquez N. Dolgert A. Fukutaki K. Fullman N. McGaughey M. (2018). Forecasting life expectancy, years of life lost, and all-cause and cause-specific mortality for 250 causes of death: reference and alternative scenarios for 2016-40 for 195 countries and territories. Lancet 392, 2052–2090. 10.1016/S0140-6736(18)31694-5 30340847 PMC6227505

[B27] Gallo Marin B. Aghagoli G. Hu S. L. Massoud C. M. Robinson-Bostom L. (2023). Calciphylaxis and kidney disease: a review. Am. J. Kidney Dis. 81, 232–239. 10.1053/j.ajkd.2022.06.011 35970430

[B28] GBD Chronic Kidney Disease Collaboration (2020). Global, regional, and national burden of chronic kidney disease, 1990-2017: a systematic analysis for the Global Burden of Disease Study 2017. Lancet 395, 709–733. 10.1016/S0140-6736(20)30045-3 32061315 PMC7049905

[B29] Hattori K. Takahashi N. Terabe K. Ohashi Y. Kishimoto K. Yokota Y. (2021). Activation of transient receptor potential vanilloid 4 protects articular cartilage against inflammatory responses via CaMKK/AMPK/NF-κB signaling pathway. Sci. Rep. 11, 15508. 10.1038/s41598-021-94938-3 34330980 PMC8324869

[B30] Hecquet C. M. Ahmmed G. U. Malik A. B. (2010). TRPM2 channel regulates endothelial barrier function. Adv. Exp. Med. Biol. 661, 155–167. 10.1007/978-1-60761-500-2_10 20204729

[B31] Hiroi T. Wajima T. Negoro T. Ishii M. Nakano Y. Kiuchi Y. (2013). Neutrophil TRPM2 channels are implicated in the exacerbation of myocardial ischaemia/reperfusion injury. Cardiovasc Res. 97, 271–281. 10.1093/cvr/cvs332 23129587

[B32] Hoenderop J. G. van Leeuwen J. P. van der Eerden B. C. Kersten F. F. van der Kemp A. W. Mérillat A.-M. (2003). Renal Ca2+ wasting, hyperabsorption, and reduced bone thickness in mice lacking TRPV5. J. Clin. Invest. 112, 1906–1914. 10.1172/JCI19826 14679186 PMC297001

[B33] Hou Y.-C. Zheng C.-M. Chiu H.-W. Liu W.-C. Lu K.-C. Lu C.-L. (2022). Role of calcimimetics in treating bone and mineral disorders related to chronic kidney disease. Pharm. (Basel) 15, 952. 10.3390/ph15080952 PMC9415417 36015101

[B34] Hruska K. A. Mahjoub M. R. (2019). New pathogenic insights inform therapeutic target development for renal osteodystrophy. Kidney Int. 95, 261–263. 10.1016/j.kint.2018.10.026 30665565

[B35] Huang C.-L. (2012). Regulation of ion channels by secreted Klotho. Adv. Exp. Med. Biol. 728, 100–106. 10.1007/978-1-4614-0887-1_7 22396165

[B36] Huang M. Wang X. Xing B. Yang H. Sa Z. Zhang D. (2018). Critical roles of TRPV2 channels, histamine H1 and adenosine A1 receptors in the initiation of acupoint signals for acupuncture analgesia. Sci. Rep. 8, 6523. 10.1038/s41598-018-24654-y 29695862 PMC5916903

[B37] Hurd L. Kirwin S. M. Boggs M. Mackenzie W. G. Bober M. B. Funanage V. L. (2015). A mutation in TRPV4 results in altered chondrocyte calcium signaling in severe metatropic dysplasia. Am. J. Med. Genet. A 167A, 2286–2293. 10.1002/ajmg.a.37182 26249260

[B38] Jiang J. Wang X. Wu X. Yu Z. (2016). Analysis of factors influencing moxibustion efficacy by affecting heat-activated transient receptor potential vanilloid channels. J. Tradit. Chin. Med. 36, 255–260. 10.1016/s0254-6272(16)30036-x 27400483

[B39] Kaur R. Singh R. (2022). Mechanistic insights into CKD-MBD-related vascular calcification and its clinical implications. Life Sci. 311, 121148. 10.1016/j.lfs.2022.121148 36336124

[B40] Khairallah P. Nickolas T. L. (2018). Management of osteoporosis in CKD. Clin. J. Am. Soc. Nephrol. 13, 962–969. 10.2215/CJN.11031017 29487093 PMC5989687

[B41] Khattar V. Wang L. Peng J.-B. (2022). Calcium selective channel TRPV6: structure, function, and implications in health and disease. Gene 817, 146192. 10.1016/j.gene.2022.146192 35031425 PMC8950124

[B42] Kidney Disease: Improving Global Outcomes (KDIGO) CKD-MBD Update Work Group (2017) (2011). KDIGO 2017 clinical practice guideline update for the diagnosis, evaluation, prevention, and treatment of chronic kidney disease-mineral and bone disorder (CKD-MBD). Kidney Int. Suppl. 7 (7), 1–59. 10.1016/j.kisu.2017.04.001 PMC6340919 30675420

[B43] Kumarasamy S. Solanki S. Atolagbe O. T. Joe B. Birnbaumer L. Vazquez G. (2017). Deep transcriptomic profiling of M1 macrophages lacking Trpc3. Sci. Rep. 7, 39867. 10.1038/srep39867 28051144 PMC5209678

[B44] Kuro-o M. (2010). Klotho. Pflugers Arch. 459, 333–343. 10.1007/s00424-009-0722-7 19730882

[B45] Lee J. Ju K. D. Kim H. J. Tsogbadrakh B. Ryu H. Kang E. (2021). Soluble α-klotho anchors TRPV5 to the distal tubular cell membrane independent of FGFR1 by binding TRPV5 and galectin-1 simultaneously. Am. J. Physiol. Ren. Physiol. 320, F559–F568. 10.1152/ajprenal.00044.2021 33615893

[B46] Lee J. J. Liu X. O’Neill D. Beggs M. R. Weissgerber P. Flockerzi V. (2019). Activation of the calcium sensing receptor attenuates TRPV6-dependent intestinal calcium absorption. JCI Insight 5, e128013. 10.1172/jci.insight.128013 31013259 PMC6629117

[B106] Lee C.-T. Ng H.-Y. Kuo W.-H. Tain Y.-L. Leung F.-F. Lee Y.-T. (2020). The role of TRPM7 in vascular calcification: Comparison between phosphate and uremic toxin. Life Sci. 260, 118280. 10.1016/j.lfs.2020.118280 32800835

[B47] Leunissen E. H. Nair A. V. Büll C. Lefeber D. J. van Delft F. L. Bindels R. J. M. (2013). The epithelial calcium channel TRPV5 is regulated differentially by klotho and sialidase. J. Biol. Chem. 288, 29238–29246. 10.1074/jbc.M113.473520 23970553 PMC3795225

[B48] Li P. Bian X. Liu C. Wang S. Guo M. Tao Y. (2018). STIM1 and TRPV4 regulate fluid flow-induced calcium oscillation at early and late stages of osteoclast differentiation. Cell Calcium 71, 45–52. 10.1016/j.ceca.2017.12.001 29604963

[B49] Lin J. Zhou S. Zhao T. Ju T. Zhang L. (2016). TRPM7 channel regulates ox-LDL-induced proliferation and migration of vascular smooth muscle cells via MEK-ERK pathways. FEBS Lett. 590, 520–532. 10.1002/1873-3468.12088 26900082

[B50] Little R. Muimo R. Robson L. Harris K. Grabowski P. S. (2011). The transient receptor potential ion channel TRPV6 is expressed at low levels in osteoblasts and has little role in osteoblast calcium uptake. PLoS One 6, e28166. 10.1371/journal.pone.0028166 22163264 PMC3226639

[B51] Liu Y. Zhang G. Zhu C. Yao X. Wang W. Shen L. (2024). The analgesic effects of Yu-Xue-Bi tablet (YXB) on mice with inflammatory pain by regulating LXA4-FPR2-TRPA1 pathway. Chin. Med. 19, 104. 10.1186/s13020-024-00975-1 39107849 PMC11302111

[B52] Loh N. Y. Bentley L. Dimke H. Verkaart S. Tammaro P. Gorvin C. M. (2013). Autosomal dominant hypercalciuria in a mouse model due to a mutation of the epithelial calcium channel, TRPV5. PLoS One 8, e55412. 10.1371/journal.pone.0055412 23383183 PMC3559602

[B53] Loukin S. Su Z. Kung C. (2011). Increased basal activity is a key determinant in the severity of human skeletal dysplasia caused by TRPV4 mutations. PLoS One 6, e19533. 10.1371/journal.pone.0019533 21573172 PMC3088684

[B54] Louvet L. Büchel J. Steppan S. Passlick-Deetjen J. Massy Z. A. (2013). Magnesium prevents phosphate-induced calcification in human aortic vascular smooth muscle cells. Nephrol. Dial. Transpl. 28, 869–878. 10.1093/ndt/gfs520 PMC3611891 23229924

[B55] Lu P. Boros S. Chang Q. Bindels R. J. Hoenderop J. G. (2008). The beta-glucuronidase klotho exclusively activates the epithelial Ca2+ channels TRPV5 and TRPV6. Nephrol. Dial. Transpl. 23, 3397–3402. 10.1093/ndt/gfn291 18495742

[B56] Luo D. Li W. Xie C. Yin L. Su X. Chen J. (2022). Capsaicin attenuates arterial calcification through promoting SIRT6-mediated deacetylation and degradation of Hif1α (Hypoxic-Inducible factor-1 alpha). Hypertension 79, 906–917. 10.1161/hypertensionaha.121.18778 35232219

[B57] Luo Y. Qiu Y. Zhou R. Zhang Y. Ji X. Liu Z. (2023). Shaoyao Gancao decoction alleviates the central hyperalgesia of recurrent NTG-induced migraine in rats by regulating the NGF/TRPV1/COX-2 signal pathway. J. Ethnopharmacol. 317, 116781. 10.1016/j.jep.2023.116781 37315643

[B58] Ma J. Zhu L. Zhou Z. Song T. Yang L. Yan X. (2021). The calcium channel TRPV6 is a novel regulator of RANKL-induced osteoclastic differentiation and bone absorption activity through the IGF-PI3K-AKT pathway. Cell Prolif. 54, e12955. 10.1111/cpr.12955 33159483 PMC7791174

[B59] Maeda R. Imura A. Nabeshima Y. (2013). Complex regulation and diverse functions of alpha-klotho. Contrib. Nephrol. 180, 25–46. 10.1159/000346777 23652548

[B60] Magagnoli L. Cozzolino M. Caskey F. J. Evans M. Torino C. Porto G. (2023). Association between CKD-MBD and mortality in older patients with advanced CKD-results from the EQUAL study. Nephrol. Dial. Transpl. 38, 2562–2575. 10.1093/ndt/gfad100 PMC10615632 37230954

[B61] Martini N. Streckwall L. McCarthy A. D. (2023). Osteoporosis and vascular calcifications. Endocr. Connect. 12, e230305. 10.1530/EC-23-0305 37698112 PMC10563638

[B62] Masuyama R. Vriens J. Voets T. Karashima Y. Owsianik G. Vennekens R. (2008). TRPV4-mediated calcium influx regulates terminal differentiation of osteoclasts. Cell Metab. 8, 257–265. 10.1016/j.cmet.2008.08.002 18762026

[B63] Matthews B. D. Thodeti C. K. Tytell J. D. Mammoto A. Overby D. R. Ingber D. E. (2010). Ultra-rapid activation of TRPV4 ion channels by mechanical forces applied to cell surface beta1 integrins. Integr. Biol. (Camb) 2, 435–442. 10.1039/c0ib00034e 20725677 PMC3147167

[B64] Menè P. Punzo G. Pirozzi N. (2013). TRP channels as therapeutic targets in kidney disease and hypertension. Curr. Top. Med. Chem. 13, 386–397. 10.2174/1568026611313030013 23432067

[B65] Michalick L. Kuebler W. M. (2020). TRPV4-A missing link between mechanosensation and immunity. Front. Immunol. 11, 413. 10.3389/fimmu.2020.00413 32210976 PMC7076180

[B66] Mittal M. Urao N. Hecquet C. M. Zhang M. Sudhahar V. Gao X.-P. (2015). Novel role of reactive oxygen species-activated Trp melastatin channel-2 in mediating angiogenesis and postischemic neovascularization. Arterioscler. Thromb. Vasc. Biol. 35, 877–887. 10.1161/ATVBAHA.114.304802 25675998 PMC4396825

[B67] Mizoguchi F. Mizuno A. Hayata T. Nakashima K. Heller S. Ushida T. (2008). Transient receptor potential vanilloid 4 deficiency suppresses unloading-induced bone loss. J. Cell Physiol. 216, 47–53. 10.1002/jcp.21374 18264976

[B68] Moe S. Drüeke T. Cunningham J. Goodman W. Martin K. Olgaard K. (2006). Definition, evaluation, and classification of renal osteodystrophy: a position statement from Kidney Disease: Improving Global Outcomes (KDIGO). Kidney Int. 69, 1945–1953. 10.1038/sj.ki.5000414 16641930

[B69] Montes de Oca A. Guerrero F. Martinez-Moreno J. M. Madueño J. A. Herencia C. Peralta A. (2014). Magnesium inhibits Wnt/β-catenin activity and reverses the osteogenic transformation of vascular smooth muscle cells. PLoS One 9, e89525. 10.1371/journal.pone.0089525 24586847 PMC3934896

[B70] Montezano A. C. Zimmerman D. Yusuf H. Burger D. Chignalia A. Z. Wadhera V. (2010). Vascular smooth muscle cell differentiation to an osteogenic phenotype involves TRPM7 modulation by magnesium. Hypertension 56, 453–462. 10.1161/hypertensionaha.110.152058 20696983

[B71] Muñoz-Castañeda J. R. Rodelo-Haad C. Pendon-Ruiz de Mier M. V. Martin-Malo A. Santamaria R. Rodriguez M. (2020). Klotho/FGF23 and Wnt signaling as important players in the comorbidities associated with chronic kidney disease. Toxins (Basel) 12, 185. 10.3390/toxins12030185 32188018 PMC7150840

[B72] Nijenhuis T. van der Eerden B. C. J. Hoenderop J. G. J. Weinans H. van Leeuwen J. P. T. M. Bindels R. J. M. (2008). Bone resorption inhibitor alendronate normalizes the reduced bone thickness of TRPV5(-/-) mice. J. Bone Min. Res. 23, 1815–1824. 10.1359/jbmr.080613 18597625

[B73] Ozcivici E. Luu Y. K. Adler B. Qin Y.-X. Rubin J. Judex S. (2010). Mechanical signals as anabolic agents in bone. Nat. Rev. Rheumatol. 6, 50–59. 10.1038/nrrheum.2009.239 20046206 PMC3743048

[B74] Parenti A. De Logu F. Geppetti P. Benemei S. (2016). What is the evidence for the role of TRP channels in inflammatory and immune cells? Br. J. Pharmacol. 173, 953–969. 10.1111/bph.13392 26603538 PMC5341240

[B75] Peacock M. (2010). Calcium metabolism in health and disease. Clin. J. Am. Soc. Nephrol. 5 (Suppl. 1), S23–S30. 10.2215/CJN.05910809 20089499

[B105] Peng J.-B. Suzuki Y. Gyimesi G. Hediger M. A. (2018). “TRPV5 and TRPV6 Calcium-Selective Channels,” in *Calcium Entry Channels in Non-Excitable Cells* . Editors J. A. Kozak and J. W. Putney (Boca Raton, FL: CRC Press/Taylor & Francis). 10.1201/9781315152592-13 30299660

[B76] Rogers M. Goettsch C. Aikawa E. (2013). Medial and intimal calcification in chronic kidney disease: stressing the contributions. J. Am. Heart Assoc. 2, e000481. 10.1161/JAHA.113.000481 24060959 PMC3835265

[B77] Smedlund K. Tano J.-Y. Vazquez G. (2010). The constitutive function of native TRPC3 channels modulates vascular cell adhesion molecule-1 expression in coronary endothelial cells through nuclear factor kappaB signaling. Circ. Res. 106, 1479–1488. 10.1161/CIRCRESAHA.109.213314 20360250

[B78] Smedlund K. B. Birnbaumer L. Vazquez G. (2015). Increased size and cellularity of advanced atherosclerotic lesions in mice with endothelial overexpression of the human TRPC3 channel. Proc. Natl. Acad. Sci. U. S. A. 112, E2201–E2206. 10.1073/pnas.1505410112 25870279 PMC4418920

[B79] Solanki S. Dube P. R. Birnbaumer L. Vazquez G. (2017). Reduced necrosis and content of apoptotic M1 macrophages in advanced atherosclerotic plaques of mice with macrophage-specific loss of Trpc3. Sci. Rep. 7, 42526. 10.1038/srep42526 28186192 PMC5301208

[B80] Song Y. Peng X. Porta A. Takanaga H. Peng J.-B. Hediger M. A. (2003). Calcium transporter 1 and epithelial calcium channel messenger ribonucleic acid are differentially regulated by 1,25 dihydroxyvitamin D3 in the intestine and kidney of mice. Endocrinology 144, 3885–3894. 10.1210/en.2003-0314 12933662

[B81] Sonou T. Ohya M. Yashiro M. Masumoto A. Nakashima Y. Ito T. (2017). Magnesium prevents phosphate-induced vascular calcification via TRPM7 and Pit-1 in an aortic tissue culture model. Hypertens. Res. 40, 562–567. 10.1038/hr.2016.188 28123180

[B82] Sun L. Yau H.-Y. Wong W.-Y. Li R. A. Huang Y. Yao X. (2012). Role of TRPM2 in H(2)O(2)-induced cell apoptosis in endothelial cells. PLoS One 7, e43186. 10.1371/journal.pone.0043186 22916222 PMC3423428

[B83] Ter Braake A. D. Tinnemans P. T. Shanahan C. M. Hoenderop J. G. J. de Baaij J. H. F. (2018). Magnesium prevents vascular calcification *in vitro* by inhibition of hydroxyapatite crystal formation. Sci. Rep. 8, 2069. 10.1038/s41598-018-20241-3 29391410 PMC5794996

[B84] van der Eerden B. C. J. Hoenderop J. G. J. de Vries T. J. Schoenmaker T. Buurman C. J. Uitterlinden A. G. (2005). The epithelial Ca2+ channel TRPV5 is essential for proper osteoclastic bone resorption. Proc. Natl. Acad. Sci. U. S. A. 102, 17507–17512. 10.1073/pnas.0505789102 16291808 PMC1297662

[B85] van der Eerden B. C. J. Koek W. N. H. Roschger P. Zillikens M. C. Waarsing J. H. van der Kemp A. (2016). Lifelong challenge of calcium homeostasis in male mice lacking TRPV5 leads to changes in bone and calcium metabolism. Oncotarget 7, 24928–24941. 10.18632/oncotarget.8779 27102152 PMC5041880

[B86] Vangeel L. Voets T. (2019). Transient receptor potential channels and calcium signaling. Cold Spring Harb. Perspect. Biol. 11, a035048. 10.1101/cshperspect.a035048 30910771 PMC6546042

[B87] van Goor M. K. C. Hoenderop J. G. J. van der Wijst J. (2017). TRP channels in calcium homeostasis: from hormonal control to structure-function relationship of TRPV5 and TRPV6. Biochim. Biophys. Acta Mol. Cell Res. 1864, 883–893. 10.1016/j.bbamcr.2016.11.027 27913205

[B88] Vazquez G. Solanki S. Dube P. Smedlund K. Ampem P. (2016). On the roles of the transient receptor potential canonical 3 (TRPC3) channel in endothelium and macrophages: implications in atherosclerosis. Adv. Exp. Med. Biol. 898, 185–199. 10.1007/978-3-319-26974-0_9 27161230

[B89] Wang J. Xiao J. Wang R. Wang D. (2023). Influencing factors of cardiac valve calcification (CVC) in patients with chronic kidney disease and the impact of CVC on long-term prognosis: a single-center retrospective study. PeerJ 11, e15569. 10.7717/peerj.15569 37404480 PMC10317020

[B90] Wang Y. Tan L. Jiao K. Xue C. Tang Q. Jiang S. (2022). Scutellarein attenuates atopic dermatitis by selectively inhibiting transient receptor potential vanilloid 3 channels. Br. J Pharmacol. 179, 4792–4808. 10.1111/bph.15913 35771623

[B91] Wei G. Xiang C. Wang H. Li X. Wu Y. Li Z. (2025). Qisheng wan decoction alleviates the inflammation of CCI rats via TRP channels. J. Ethnopharmacol. 338, 118990. 10.1016/j.jep.2024.118990 39490711

[B92] Wen H. Gwathmey J. K. Xie L.-H. (2020). Role of transient receptor potential canonical channels in heart physiology and pathophysiology. Front. Cardiovasc Med. 7, 24. 10.3389/fcvm.2020.00024 32158769 PMC7052113

[B93] Wolf M. T. F. An S.-W. Nie M. Bal M. S. Huang C.-L. (2014). Klotho up-regulates renal calcium channel transient receptor potential vanilloid 5 (TRPV5) by intra- and extracellular N-glycosylation-dependent mechanisms. J. Biol. Chem. 289, 35849–35857. 10.1074/jbc.M114.616649 25378396 PMC4276853

[B94] Yan Y.-F. Feng Y. Wang S.-M. Fang F. Chen H.-Y. Zhen M.-X. (2024). Potential actions of capsaicin for preventing vascular calcification of vascular smooth muscle cells *in vitro* and *in vivo* . Heliyon 10, e28021. 10.1016/j.heliyon.2024.e28021 38524547 PMC10958412

[B95] Yang M. Fang J. Liu Q. Wang Y. Zhang Z. (2017). Role of ROS-TRPM7-ERK1/2 axis in high concentration glucose-mediated proliferation and phenotype switching of rat aortic vascular smooth muscle cells. Biochem. Biophys. Res. Commun. 494, 526–533. 10.1016/j.bbrc.2017.10.122 29079194

[B96] Yang N. Deng J. Xu H. Dai H. Jin H. Shao H. (2024). Anti-atopic dermatitis effect of fraxinellone via inhibiting IL-31 *in vivo* and *in vitro* . Heliyon 10, e35391. 10.1016/j.heliyon.2024.e35391 39170490 PMC11336620

[B97] Zelt J. G. E. McCabe K. M. Svajger B. Barron H. Laverty K. Holden R. M. (2015). Magnesium modifies the impact of calcitriol treatment on vascular calcification in experimental chronic kidney disease. J. Pharmacol. Exp. Ther. 355, 451–462. 10.1124/jpet.115.228106 26487689

[B98] Zeng H. Zhang Z. Zhou D. Wang R. Verkhratsky A. Nie H. (2025). Investigation of the anti-inflammatory, anti-pruritic, and analgesic effects of sophocarpine inhibiting TRP channels in a mouse model of inflammatory itch and pain. J. Ethnopharmacol. 337, 118882. 10.1016/j.jep.2024.118882 39366497

[B99] Zhang K. Zhang Y. Feng W. Chen R. Chen J. Touyz R. M. (2017). Interleukin-18 enhances vascular calcification and osteogenic differentiation of vascular smooth muscle cells through TRPM7 activation. Arterioscler. Thromb. Vasc. Biol. 37, 1933–1943. 10.1161/ATVBAHA.117.309161 28860220

[B100] Zhang Y. Ying F. Tian X. Lei Z. Li X. Lo C.-Y. (2022). TRPM2 promotes atherosclerotic progression in a mouse model of atherosclerosis. Cells 11, 1423. 10.3390/cells11091423 35563730 PMC9103947

[B101] Zhong G. Long H. Chen F. Yu Y. (2021). Oxoglaucine mediates Ca2+ influx and activates autophagy to alleviate osteoarthritis through the TRPV5/calmodulin/CAMK-II pathway. Br. J. Pharmacol. 178, 2931–2947. 10.1111/bph.15466 33786819

[B102] Zhou D.-M. Sun L.-L. Zhu J. Chen B. Li X.-Q. Li W.-D. (2020). MiR-9 promotes angiogenesis of endothelial progenitor cell to facilitate thrombi recanalization via targeting TRPM7 through PI3K/Akt/autophagy pathway. J. Cell Mol. Med. 24, 4624–4632. 10.1111/jcmm.15124 32147957 PMC7176881

[B103] Zhu J.-G. Chen J.-B. Cheng B.-C. Lee C.-H. Long G. Chien Y.-S. (2018). Association between extreme values of markers of chronic kidney disease: mineral and bone disorder and 5-year mortality among prevalent hemodialysis patients. Blood Purif. 45, 1–7. 10.1159/000478972 29161692

[B104] Zou Z.-G. Rios F. J. Montezano A. C. Touyz R. M. (2019). TRPM7, magnesium, and signaling. Int. J. Mol. Sci. 20, 1877. 10.3390/ijms20081877 30995736 PMC6515203

